# Antidepressants and Suicide Risk: A Comprehensive Overview

**DOI:** 10.3390/ph3092861

**Published:** 2010-08-30

**Authors:** Maurizio Pompili, Gianluca Serafini, Marco Innamorati, Elisa Ambrosi, Gloria Giordano, Paolo Girardi, Roberto Tatarelli, David Lester

**Affiliations:** 1 Department of Neurosciences, Mental Health and Sensory Functions, Suicide Prevention Center, Sant’Andrea Hospital, Sapienza University of Rome, Via Grottarossa 1037, 00189, Rome, Italy; E-Mails: gianluca.serafini@uniroma1.it (G.S.); innamorati.marco@libero.it (M.I.); ambrosi.e@libero.it (E.A.); giordano_gloria@libero.it (G.G.); paolo.girardi@uniroma1.it (P.G.); roberto.tatarelli@uniroma1.it (R.T.); 2 McLean Hospital – Harvard Medical School, 115 Mill Street, Belmont, MA 02478, USA; 3 The Richard Stockton College of New Jersey, P.O. Box 195 Pomona, NJ 08240, USA; E-Mail: david.lester@richardstockton.edu

**Keywords:** suicidal risk, antidepressants, pharmacotherapy, younger patients

## Abstract

The annual worldwide suicide rate currently averages approximately 13 per 100,000 individuals per year (0.013% per year), with higher average rates for men than for women in all but a few countries, very low rates in children, and relatively high rates in elderly men. Suicide rates vary markedly between countries, reflecting in part differences in case-identification and reporting procedures. Rates of attempted suicide in the general population average 20–30 times higher than rates of completed suicide, but are probably under-reported. Research on the relationship between pharmacotherapy and suicidal behavior was rare until a decade ago. Most ecological studies and large clinical studies have found that a general reduction in suicide rates is significantly correlated with higher rates of prescribing modern antidepressants. However, ecological, cohort and case-control studies and data from brief, randomized, controlled trials in patients with acute affective disorders have found increases, particularly in young patients and particularly for the risk of suicide attempts, as well as increases in suicidal ideation in young patients. whether antidepressants are associated with specific aspects of suicidality (e.g., higher rates of completed suicide, attempted suicide and suicidal ideation) in younger patients with major affective disorders remains a highly controversial question. In light of this gap this paper analyzes research on the relationship between suicidality and antidepressant treatment.

## 1. The Complex Challenge of Suicide

The annual worldwide suicide rate currently averages approximately 13 per 100,000 individuals per year (0.013% per year), with higher average rates for men than women in almost all countries, low rates in children, and relatively high rates in elderly men [1,2,3,4]. Suicide rates vary markedly among regions of the world, countries and locales, in part reflecting differences in case-identification and reporting procedures [2,5]. Estimated rates of attempted suicide are much less reliable than for completed suicide. Rates of attempted suicide in the general population average 20–30 times higher than rates of completed suicide, but are probably under-reported. Suicide attempts range widely in method and seriousness of intent [6,7]. The ratio of attempts/suicides in clinical samples of mood-disorder patients is much lower, suggesting a greater lethality of suicidal behaviors [8]. Most cases of suicide (81%–95%) in the general population involve psychiatric illnesses, nearly half of which (48.5%) are mood disorders [9]. Internationally, the overall average suicide rate has been rising gradually over the past half-century, with increases reported in about half of the nations, mainly among men, and moderate decreases found in the other half, including Northern Europe and North America. These improvements could reflect improved diagnosis and treatment of mood disorders during the same period [4,10,11,12,13]. The risk of completed suicide within five years is twice as high among men than women (8.3% *vs*. 4.3%), as expected, and most occurred within a year of follow-up by a psychiatric hospital [14]. The ratio of attempts to subsequent suicides (A/S) also was half as great in men as in women (12:1 *vs*. 23:1), consistent with the greater lethality of suicide actions in men. The risk of subsequent suicide was higher in men below the age of 35 versus above the age of 35 (9.7% *vs*. 7.0%), but higher in women older than this age than those below this age (6.0% *vs*. 2.5%). Accordingly, the relative risk among younger men was four-times higher than in younger women (9.7% versus 2.5%), whereas the relative risk in older men and women did not differ much (7.0% versus 6.0%), indicating that the lethality of suicidal behavior in women over age 35 approaches that of men of similar age. Suicide rates are surprisingly high among persons diagnosed with anxiety disorders [15,16]. 

Severe anxiety may accompany suicidal behavior [17], but the evidence that anti-anxiety medications reduce suicidal risk in anxiety disorder patients is very limited [16]. The general risk factors for suicide include the presence of a major mood disorder with hopelessness, especially when co-occurring with alcohol or substance abuse [2,6,9,18]. The risk is especially high among young, impulsive-aggressive and older unmarried men [2,3]. The risk of suicide in bipolar patients continues to be high, despite the introduction of a growing number of new treatments. Tondo, Lepri, and Baldessarini [19] suggested that the risk of dying by suicide among bipolar disorder patients is nearly 10%, assuming potential risk-exposures of 60 years or more. More than one-third of all suicidal acts occurred within the first year from illness-onset, and more than half within the first five years of illness, indicating the need for early diagnosis and intervention in major mood disorders. Approximately three-quarters of the unresolved morbidity among bipolar disorder patients is a result of depression [20,21,22,23], and this, no doubt, encourages high rates of utilization of antidepressants as a treatment option, even though such treatment is neither approved by regulatory bodies, nor well supported by available clinical research as either effective or safe in bipolar patients [24,25]. Furthermore, antidepressant treatments are associated in some patients with dysphoric-mixed-agitated or psychotic states that can increase suicidal risk [26,27,28]. The effects of treatments on suicidal risk have been a topic of growing research interest in recent years, but evidence that most modern psychiatric treatments reduce long-term suicide risks is very limited [2,3,29,30,31]. In view of this gap, the present chapter will systematically review whether the use of antidepressants is associated with benefits in terms of completed or attempted suicide and whether the increasing use of SSRIs is associated with benefits in terms of decreased rates of suicide, with the aim of generating evidence-based suggestions for clinicians.

In order to provide an overview of research antidepressants and suicide risk, we performed careful MedLine, Excerpta Medica, PsycInfo and Index Medicus searches to identify all papers and book chapters in English during the period 1965–2010. The search terms used were “suicide”, “suicidal”, “suicidality” and all suicide-related terms, “antidepressants”, “antidepressant agents”, “selective serotonin reuptake inhibitors (SSRI)”, “Serotonin-Norepinephrine Reuptake Inhibitors (SNRI)” “noradrenergic and specific serotonergic antidepressants (NaSSA)”, “selective noradrenaline reuptake inhibitor (NaRI),” “Norepinephrine and Dopamine Reuptake Inhibitor (NDRI)” “tricyclics (TCA)”, and “Monoamine oxidase inhibitor (IMAO)”. Where a title or abstract seem to describe a study eligible for inclusion, the full article was obtained and examined to ascertain its relevance based on the inclusion criteria. Any discrepancies between the two reviewers who, blind to each other, examined the studies for possible inclusion, were resolved by consultations with senior authors. The combined search strategies yielded over 500 abstracts. After a complete analysis of the abstracts, 200 full-text articles were reviewed. Approximately 130 studies met our inclusion criteria and were included, focusing on the most recent studies for the present review.

## 2. Antidepressants and Suicidal Risk

Only recently has the effect of antidepressant treatments on suicidal behavior and the increased overall mortality associated with major psychiatric disorders become a focus of research interest [32,33,34]. A highly controversial question is whether antidepressant treatment modifies the risk of various aspects of “suicidality” among patients with major depressive disorders [35,36,37,38,39,40,41], especially whether the selective serotonin reuptake inhibitors (SSRIs) may cause worsening of suicidal thoughts in vulnerable people [42,43]. 

In 2005, a systematic review of published randomized controlled trials comparing SSRIs with other active treatments or placebo found an almost 2-fold increase in the odds of fatal and nonfatal suicide acts among those exposed to SSRIs [44]. However, no increased risk was observed when only fatal suicide acts were included. Among adolescents, a recent study found that SSRIs utilization significantly increased the risk of completed or attempted suicide [45] whereas, among adults, SSRI use significantly decreased the risk of completed or attempted suicide and, among people aged 65 or more years, the use of SSRIs had a significant protective effect [45]. 

Another systematic review by Gunnell *et al*. [46] compared the use of SSRIs and placebo in adults with depression and other clinical conditions and showed no evidence of increased risk of completed suicide and only weak evidence of increased risk of non-fatal self-harm. More recently, the U.S. Food and Drug Administration (FDA) performed a meta-analysis of individual patient data from 372 randomized placebo-controlled trials of antidepressants, with a total of nearly 100,000 patients, and reported that suicidal behavior was strongly related to age [47]. The risk associated with antidepressant use relative to placebo was increased among patients aged 25, or younger whereas it was reduced among patients aged 65 or more. The risk among adult patients aged 25–64 years was not significant, but it was reduced when suicidal ideation and behavior were considered together [48]. Based on these findings, in 2007 the FDA ordered that all antidepressant drugs carry an expanded black-box warning on their label that included information about the increased risk of suicidal behavior in young adults aged 18–24 years [49,50]. 

O'Leary *et al*. [51] reviewed suicide rates in affective disorder patients and the impact of the electroconvulsive therapy (ECT) era *vs.* the antidepressant era in 75 follow-up studies. Suicide accounted for more than 12% of all deaths in samples in which 40% or more of patients had died. They found that suicide rates decreased with longer follow-up periods; the mean suicide rate was 6.3 per 1,000 for the pre-treatment era, 5.7 per 1,000 for the ECT treatment era (1940 to 1959) and 3.3 per 1,000 for the antidepressant treatment era (from 1960 on). They concluded that the utilization of ECT and antidepressants may have contributed to this decrease, although other variables may be involved.

One important limitation to take into account is that the trials included by FDA were not primarily designed to measure any particular suicidal behavior. Instead they used a composite outcome of suicidal ideas, preparatory acts, suicide attempts and deaths by suicide [48]. Additionally, of all suicidal events that were reported, less than 30% were serious suicide attempts or deaths. Furthermore, considering that suicidality was self-reported rather than observed by others in the majority of the clinical trials, it is possible that antidepressant treatment, particularly in younger subjects enhanced communication about suicidality, which may promote more articulated and open thoughts and behaviours. 

Most of studies on the effect of antidepressant therapy on the risk of suicide have a number of other additional limitations. First, observational studies have limited ability to adjust for baseline differences and are prone to bias and confounding. Also, among adolescents, SSRI treatment is often reserved for the very severe cases, and so the higher suicide rate might be confounded by severity, that is, adolescents who received SSRIs might have been more severely depressed (or more suicidal) than adolescents who did not receive SSRIs. In contrast, among adults, SSRIs may be prescribed for both severe and less severe cases, and confounding might be less important. Only a few studies have considered confounding factors such as the severity of the illness for which the antidepressant medication was prescribed. 

Barbui *et al* [45] argued that, although re-analyses of randomized studies, including the FDA study, reported similar differences between antidepressants [50,52,53], it is not clear why the use of only certain antidepressants, such as paroxetine and venlafaxine, increases the risk of suicide more than others. They suggested that new evidence such as head-to-head comparisons are needed to test properly the long-term efficacy and safety of those medications in clinical practice, particularly in younger patients. 

In Europe, the European Medicines Agency (EMEA) has ruled that SSRIs and selective serotonin and norepinephrine reuptake inhibitors (SNRIs) should not be prescribed for depression in children and adolescents under the age of 18. The Committee for the Safety of Medicines in the UK has advised that the balance of risks and benefits for the treatment of depression in the pediatric population is unfavourable for most SSRIs.

In recent years, to explore this issue, many different types of studies have been performed, including ecological analyses, observational studies, case-control studies and randomized clinical trials. 

## 3. The Relationship between Antidepressants and Suicide as Reported in Ecological Studies

The evidence that antidepressant treatment yields reductions in the risk of suicides or attempts at the national level is controversial. Research suggests that in most of the Nordic countries and in the USA, the use of modern antidepressants over the past decade in clinical practice was associated with generally moderate decreases in overall suicide rates, varying by sex and age groups [10,11,13,54,55,56,57,58,59,60,61]. However, in the USA and some Nordic countries, similar trends regarding a reduction of suicide rates were also reported a decade before the introduction of fluoxetine, the first clinically successful modern antidepressant [13,61]. 

Sebestyen *et al*. [62] conducted a time trend analysis investigating the correlation between the trends of antidepressant prescriptions and suicide rates and a seasonality index (the extent of the seasonality in suicide rates) in Hungary between 1998 and 2006. They found that increasing antidepressant utilization was associated with a significantly decreased seasonality of suicides only among males. 

Recently Baldessarini *et al*. [13] reported that only nine of 29 ecological studies reported significant inverse correlations between increased use of modern antidepressants and declining trends in suicide rates. Six found no relationships and 14 found inconsistent correlations over sex or age subgroups. 

Data from 78 reporting countries indicated that only about half (54%) experienced decreases in their suicide rates between the 1950s (when antidepressants were not available) and the early 2000s (a decade after introduction of modern antidepressants); 46% reported increases in their suicide rates. Similar inconsistencies in the suicide rate trends were found in each region (Western and Eastern Europe, Latin America and Asia). The distribution of decreasing and increasing suicide rates of nations between 1990 and the early 2000s was similar [4]. These data suggest that trends in suicide rates are a highly complex phenomenon which reflects the existence of many contributing factors. 

Kalmar *et al*. [63] analyzed annual antidepressant prescription rates and suicide rates of about 10 million inhabitants between 1999–2005 by age and gender in Hungary. They reported that the suicide rate was inversely related to the increased utilization of antidepressants in both males and females. The antidepressant prescription rate was also related to suicide rate even after controlling for many variables such as the divorce rate and the unemployment rate, although not after controlling for the alcohol consumption rate.

Ecological studies should be considered in light of several limitations. First, it is possible that improvements in public health, changes in legal, cultural or social prohibitions against suicide and improvements in identifying and reporting suicides might be associated with higher suicide rates despite the increased access to modern psychopharmacological treatment. Socioeconomic factors may also contribute to the complex findings from the ecological studies, as well as access to clinical services and social support. Finally, all correlational studies suffer from a fundamental inability to relate medication exposure or other specific factors *causally* to suicidal behavior at the level of individuals [64]. 

Ecological studies cannot indicate a direct, causal relationship between antidepressant treatment and reduced suicidal risk at the level of individual persons, and further research with less ambiguous methods are needed.

## 4. The Relationship between Antidepressants and Suicide as Reported in Cohort and Case-Control Studies

Data from the cohort and case-control studies suggest that the utilization of antidepressants markedly increased during the 1990s, but in recent years their utilization has decreased as a result of concerns regarding the emergence of suicidality during antidepressant treatment. 

Recently, Schneeweiss *et al*. [65] conducted a cohort study of users of antidepressant agents in a population of 287,543 adults aged 18 years and older after the initiation of antidepressant treatment. They found rates of suicidal behavior ranging from 4.41 per 1,000 person-years to 9.09 per 1,000 person-years, and the majority of events occurred in the first six months after treatment initiation. Compared with selective serotonin reuptake inhibitors, serotonin-norepinephrine reuptake inhibitors, tricyclic agents, and other newer agents showed a similar risk. They supported the US Food and Drug Administration's decision to treat all antidepressants alike in their advisory, and they suggested that clinicians should be vigilant in monitoring patient behavior after initiating treatment with antidepressant agents.

Bailly [66] suggested that, relative to placebo, SSRIs are efficacious for paediatric patients with affective disorders but may be associated with a modest increase in the risk of occurrence of suicidal ideation and behaviour. However, he argued that their utilization is also associated with a significant decrease in the suicide rates in children and adolescents, presumably because of their efficacy, compliance, and low toxicity in overdose. Recent research suggests that antidepressants may be protective against early readmission after hospitalization for suicide attempts or ideation [67]. These data, taken together, indicates that SSRIs can improve adolescent depression better than placebo [68], although the magnitude of the antidepressant effect is 'small to moderate' because of a high placebo response.

Considerable recent attention and wide-scale interventions by regulatory authorities have changed drug usage patterns. In a 9-year cohort study 20,906 children who were placed on antidepressant therapy, Schneeweiss *et al*. [69] found that 266 attempted suicide and three completed suicide, with no meaningful differences in the rates in patients given different SSRIs. They supported the decision of the Food and Drug Administration to include all antidepressants in the black box warning regarding the potentially increased suicidality risk for children and adolescents who begin the use of antidepressants.

The same authors [69] suggested that treatment decisions should be based on the medication’s efficacy, and clinicians should be vigilant in monitoring patients after initiating therapy with any antidepressant. In fact, in a cohort study of 287,543 adults aged 18 years and older who used antidepressants, they found that most suicidal events occurred in the first six months after treatment initiation. There was no clinically meaningful variation in the rates of suicide and suicide attempt between the different antidepressants. 

In conclusion, the increased risk of suicidality in adolescents, compared to adults, is small, but consistent across most studies, although epidemiological studies do not support a clear relationship between use of antidepressants and the completed suicide rate [68]. Reeves and Ladner [70] suggested that antidepressant-induced suicidality was an uncommon occurrence but also a legitimate phenomenon. Close monitoring is needed, and adequate follow-up care should be provided for patients after initiation of a new antidepressant.

Other sources of data about this controversial topic include large cohorts of depressed patients from general practice or health-maintenance organizations, and relatively large, case-control comparisons of subgroups varying in exposure to antidepressants. Such studies include over one million, mainly clinically depressed, subjects in 17 reports [71,72,73,74,75,76,77,78,79,80,81,82,83,84,85,86,87]. These studies have yielded inconsistent findings. Many (n = 11) were designed to test for greater suicidal risk with SSRI antidepressants compared to other agents or to non-treatment. Nine of these 11 studies did not find evidence of greater suicidal risk associated with antidepressant treatment, and a majority of the other studies (4 out of the 6) found decreased or no difference in suicidal risk with more antidepressant treatment. 

A few of these studies found greater suicidal risk with more antidepressant treatment in general or, specifically, with the greater use of SSRIs and other modern antidepressants. By age group, they found small increases among adolescents [83,87] and larger increases in elderly patients [82], but fewer completed suicides and more attempts [85]. 

However, data from these studies are severely compromised by confounding from morbidity or by indication. As already specified, medical treatments, in general, are more likely to be given to more severely ill patients who are, therefore, at higher risk of suicide. Treatment with a low risk of toxic effects from acute overdosing with the SSRIs means that they are more likely to be prescribed than are more toxic agents (such as the tricyclic antidepressants) for patients at increased risk for suicide. Additionally, the nonrandomized, clinically-selected treatments in such studies may severely distort the associations observed between greater suicidal risk and the use of some treatments. 

## 5. The Relationship between Antidepressants and Suicide as Reported in Randomized Controlled Trials (RCTs)

RCTs should be the most reliable source of data on the effects of antidepressant treatment on suicidal risk. However, individual trials are unlikely to include enough patients with identified suicidal actions to allow testing for treatment effects, especially since being acutely suicidal is usually a criterion for exclusion from such trials. 

Meta-analytic methods by combining the results of many studies may yield substantial numbers of suicide attempts and even completed suicides. Rates of suicide attempts, and even of completed suicides, may be as high in controlled trials as in clinical samples of patients with major depressive disorder. Suicide rates taken from large meta-analyses of modern and older antidepressants or placebos averaged 862 per 100,000 per year [88,89,90], or 66-times above the average international general population suicide rate of 13 per 100,000 per year [4]. In clinical samples of severely ill major depression patients, usually hospitalized patients, the standardized mortality ratio (SMR) for suicide compared to the general population was 20-fold higher [15], and among less severely ill depressed patients this risk ratio may be about three-times higher [91]. Such high observed rates of suicidal behaviors in RCTs reflect exaggeration arising from annualizing rates based on relatively brief exposure times (typically 8–12 weeks) in most trials of patients with acute depression and early in the course of treatment. 

There have been several recent reports based on pooling data on rates of completed and attempted suicide from large numbers of RCTs and subjects involving modern antidepressants, older standard agents and placebos [37,44,46,48,88,89,90,92,93,94,95,96]. With the exception of one report which found a significant excess of suicidal acts during treatment with SSRI antidepressants [44], other meta-analyses have found only minor differences in rates of suicidal behaviors between depressed patients treated with an SSRI, other antidepressants, or a placebo, with an overall tendency toward an apparently greater rate with antidepressants. It was assumed that the trials included in the meta-analyses were well-randomized and with well-balanced exposures in both the drug and placebo groups throughout the duration of the trials. 

In a meta-analysis, Baldessarini *et al*. [37] reported that the overall pooled Rate Ratio (RR) and its 95% confidence interval (CI) was 1.42, compared to the null value of RR=1.0 whereas Fergusson *et al*. [44] reported a relatively large RR. The authors concluded that more than 600 depressed patients would have to be exposed to an antidepressant drug for approximately two months (or about 100 treated for a year), early in the course of an episode of acute major depressive disorders, when the suicidal risk is particularly high, to yield one excess suicide attempt, and over 4,500 depressed patients to yield one excess suicide over the number associated with untreated major depression. 

An FDA review [48] analyzed all available data from 386 controlled trials of modern antidepressant drugs involving 112,875 patients with major depressive or other disorders. Most of the identified suicidal events involved suicidal ideation, but were of uncertain clinical significance. Reported instances of suicides (0.013%) and attempts (0.198%) may be low due to the relatively brief exposure times, which averaged only two months. Among adult patients diagnosed with major depressive disorder, the observed suicide rate, based on estimated exposure-times averaging eight weeks, was 77 per 100,000 per year, or only six-times above the general population rate of [4], compared to suicide rates of 862 found in similar controlled trials of modern antidepressants as already noted [88,89,90]. The stratifying by age-groups analysis suggested an increased risk of broadly defined “suicidality” (mainly ideation) with modern antidepressants versus placebo at younger ages and possible beneficial effects in older patients. It is important to note that, based on these data, completed suicide and attempted suicide were rare.

These findings suggest an increased risk of presumably new suicidal ideation with SSRIs and other modern antidepressants over placebo among children, adolescents and young adults up to age 24 years and reinforced the initial findings in meta-analyses involving children and adolescents by the UK drug regulatory agency [97] and the FDA [94,96]. 

The data fail to support an expected overall reduction of risk of suicidal acts during treatment with antidepressants of all types, and this suggests that the risk of suicide attempts and perhaps completed suicides may be greater during treatment with antidepressants of various types. However, although many trials involving patients with depressive disorders found substantially larger reductions in the average ratings of suicidal ideation with antidepressants of various types than with placebo [90,92,98,99,100], unfortunately these trials reported subjective data, usually incidental to the main purpose of the study, and based on post-hoc assessments of individual items on standard depression symptom-rating scales (e.g., Hamilton item 3 or Montgomery-Åsberg item 10). 

RCTs are limited by the potential unreliability of essentially incidental, passive reporting of suicidal thoughts or behaviors based on currently typical “adverse event reporting” systems. In addition, the relatively short duration of most trials of treatments for acute depression may not provide an adequate evaluation of the effects of treatment on suicidal behaviors which are rare, comparable to the long-term trials available for clozapine, lithium and anticonvulsants. Another potential source of bias is that initial randomization in RCTs can break down during the conduct of trials. Premature, and potentially non-random, dropping out, usually a result of the lack of any perceived benefit or the emergence of intolerable side effects of the treatment, is routinely found in most RCTs. Observed rates of “suicidal events” are rarely corrected for the actual time of exposure of individual patients to specific treatments. Such correction for actual adverse event rates (events/persons/weeks) may be important if actual exposures are not closely matched across trial treatment arms, particularly when rare events such as completed and attempted suicides are involved [101]. 

After reviewing all of the data, the FDA demanded warnings about the possible increased of risk of suicidal ideation and perhaps behaviors during the treatment of juvenile and young adult patients with SSRIs and other modern antidepressants. This decision was based on at least four controlled trials [102], one of which was a very large federally-funded trial with an unusually large separation of responses to the drug versus placebo [103]. In 12 relatively large trials of SSRIs in juvenile patients with major depression, the relative rate of responding to the drug relative to the placebo (RR) averaged 1.23 and, in 14 much smaller trials of TCAs, the pooled RR of 1.15 was of similarly modest magnitude and statistically nonsignificant [102]. The conclusion about the possible worsening of suicidality in young antidepressant-treated patients implies that there are clinically developmental differences in affective and behavioral responses to antidepressants that require further study, especially with respect to the risks of actual suicidal behaviors.

## 6. The Relationship between Antidepressants and Suicide in Different Countries

In recent years, antidepressant prescribing has increased markedly in many developed countries, especially after the introduction of the SSRIs in the early 1990s. This might reflect the better recognition, treatment and prescribing for depression and also the advantage that these new medications rarely result in lethal overdoses. The associations between antidepressants and suicide rates differ by country. In Italy, Barbui *et al*. [104] described the impact of regulatory changes on antidepressant consumption in Italy from 1988 to 1996, examining whether the introduction of SSRIs was associated with changes in the overall suicide rates [104]. Data on suicide, available from 1988 to 1994, showed that, in the seven-year period during which SSRI use rose, male suicide rates increased from 9.8 to 10.2 per 100,000 inhabitants while females suicide rates declined from 3.9 to 3.2. The authors concluded that, in Italy, there was not any dramatic reduction in the overall suicide rates as a result of the increasing prescribing of SSRIs. Guaiana *et al*. [105] investigated the impact of the increasing use of SSRIs and newer antidepressants on suicide rates and reported that, in Italy from 1983 to 2000, the use of tricyclic antidepressants remained stable while the use of SSRIs and newer agents dramatically increased. Suicide rates for males decreased from 1955 to 1974 and subsequently increased, reaching a peak in 1985 and then declining. In females, suicide rates remained substantially stable until 1978. Suicide by poisoning dropped by nearly 50% from 1986 to 2000. Long-term analysis did not indicate that the increase in antidepressant prescribing was accompanied by a reduction in the suicide rate. Furthermore, in Italy, newer antidepressants had no impact on the total number of admissions for depression, nor on the proportion of all admissions.

In Sweden, Carlsten *et al*. [106] performed a time-series analysis using a two-slope model to compare suicide rates before and after the introduction of the SSRIs. Antidepressant sales increased in men from 4.2 DDD per 1,000 inhabitants per day during 1977–1979 to 21.8 in 1995–1997 and in women from 8.8 to 42.4 between the same periods. A particularly rapid acceleration in antidepressant sales was observed during the years 1993–1996. Between the two periods, suicide rates decreased in men by 30.9% and by 34.0% in women. The slope of suicide rates significantly changed after 1990, corresponding to 348 fewer suicides than expected during 1990-1997. This study showed a statistically significant change in the slope in suicide rates among men and women that coincided with the introduction of the SSRIs in Sweden. However, the advent of SSRIs may explain only part of the declining suicide trend because this trend started years before these drugs were introduced. Isacsson [10] found that suicide rate decreased by 19% in parallel with the increased use of antidepressants in Sweden, Denmark, Norway and Finland. Moreover, in Sweden there was no demographic group with regard to age, gender or county in which the suicide rate decreased in the absence of an increased use of antidepressants. Thus, the author concluded that the increased use of antidepressants agents appeared to be one of the contributing factors to the decrease in the suicide rate. Ohberg *et al*. [107] found that, in Finland from 1990 to 1995, the total suicide rate decreased from 30.3 per 100,000 inhabitants per year to 27.2. The total consumption of antidepressants drug more than doubled from 1990 to 1995. The authors concluded that the increased use of SSRIs in Finland coincided with a significant decline in suicide mortality. However, suicides using antidepressants showed an upward trend.

Rihmer reported that, in Hungary, where suicide rates were extremely high, the suicide rate steadily declined from 45.9 per 100,000 inhabitants per year in 1984 to 32.1 in 1998 [108,109,110]. This decrease occurred together with the implementation of a new system of psychiatric care in which the number of outpatient psychiatric departments increased between 1982 and 1998, the number of psychiatrists increased between 1986 to 1998, and the number of emergency telephone services increased during the same period. Antidepressant consumption, mainly SSRIs, grew approximately fivefold from 1984 to 1998. In the same period there was a sixfold increase in unemployment, a 25% rise in official estimates of alcoholism rates and a 21% increase in divorce, and so the decrease in suicide rates was remarkable. However, Rihmer cautioned that suicide rates are affected by many factors, and the association between improved pharmacotherapy for depression and decreased suicide mortality is not easily demonstrated.

Hall and colleagues [111] found that, in Australia between 1991 and 2000, the suicide rate decreased markedly in old men and women and increased in young adults, especially in young men, yielding a substantially stable total suicide rate. Exposure to antidepressants was higher for women than men in all age groups and increased markedly for both men and women, with the largest increases among older adults. A significant negative association was observed between antidepressant consumption and suicide in women but not in men. However, among both men and women the largest declines in suicide occurred in the age groups with the highest exposure to antidepressants. 

Grunebaum *et al*. [11] showed that, in the United States from 1985 to 1999, the decline in the national suicide rate appeared to be associated with a greater use of non-tricyclic antidepressants. Suicide rates were already declining before 1985 in the United States, especially in females [112,113]. In an ecological analysis over counties in the USA, Gibbons and colleagues [55] found that the overall association between antidepressant prescriptions and the suicide rate was not significant for prescriptions for SSRIs, but the prescription of other new-generation non-SSRI antidepressants (nefazodone hydrochloride, mirtazapine, bupropion hydrochloride, and venlafaxine hydrochloride) was associated with lower suicide rates (both within and between counties). Among adolescents, exposure to paroxetine and venlafaxine were significantly associated with increased risk [83,85], and so the effect of age seems to be critical [45].

In the UK, Davis *et al*. [114] studied whether a higher suicide risk, defined as previous suicide attempts or suicidal ideation, influenced the choice of antidepressant prescribed. They found that a risk of suicide did not prevent the prescription of venlafaxine, and that venlafaxine therapy is considered to be acceptable and is appropriately managed. 

Also in the UK, Gunnell *et al*. [115] reported a time-series analysis which documented an increase in young male suicide in England and Wales in the last 30 years, in parallel with a rise in a number of risk factors for suicide in this age group. In people aged over 60 an association was found between increases in the gross domestic product, antidepressant prescribing and other measures of improved healthcare provision and a decline in suicide. Also in England, Morgan *et al*. [119] found that the increased prescribing of antidepressants, a proxy measure of improved diagnosis and treatment of depression in primary care, was accompanied by lower suicide rates.

Kasper *et al*. [116], in a longitudinal analyses of pooled data from 15 placebo-controlled, randomized, double-blind, short-term trials of mirtazapine conducted in Austria, found that mirtazapine was associated with statistically significantly lower suicidality risk compared to placebo.

In the pediatric population, higher SSRI prescription rates have been found to be associated with lower suicide rates in children and adolescents [56]. Similar findings were reported by Olfson and colleagues [83] in an extensive analysis of prescription data in the USA. However, all these positive finding may reflect not only the efficacy of SSRIs, but also better compliance and quality of mental health care and a low toxicity in the event of a suicide attempt by overdose. 

In Northern Ireland, Kelly *et al*. [117] observed that, in the younger age groups, there was no association between antidepressant prescribing and suicide, while for the older age groups increased antidepressants prescribing was associated with a reduction in the suicide rate over a period of 10 years. However, a subsequent analysis of British data suggested that the decline in suicide rates happened before the increase in prescribing. Moreover, rises in antidepressant prescribing did not consistently coincide with clear changes in suicide trends [118]. 

In Iceland sales of antidepressants increased from 1975 to 2000, but suicide rates fluctuated during the period of 1950-2000 and did not show any definite trend [54].

Finally, in Israel, the prescribing of antidepressants, particularly of SSRIs, increased 2.6-fold between 1998 and 2002. An overall reduction in suicides was reported in association with this increased rate of antidepressant prescription [120], but was statistically significant only for elderly men.

## 7. Possible Explanations for the Lack of Effect on Suicidal Risk during Antidepressant Treatment and Clinical Implications

Several explanations for the eventual lack of overall beneficial effect on the rate of completed and attempted suicide during antidepressant treatment have been proposed, including limitations to the clinical effectiveness of antidepressants, particularly in younger age-groups, and for some features of depressive illnesses that may be particularly relevant to suicidal risk, as well as technical limitations of the studies of antidepressant treatment and suicide [36]. An additional possibility is that antidepressants may have both beneficial and adverse effects, with a net zero average impact on suicidal rates. 

These conclusions are supported by the long tradition of clinical observation, including the proposal that temporal separation of the relatively early energizing effects of mood-elevating drugs from later benefits on the anhedonic features of depression might increase the risk of suicidal behaviors during recovery from acute major depressive episodes [121]. Additionally, there are a series of clinical observations of newly emerging aggressive and suicidal preoccupations or behaviors among patients being treated with antidepressants. Whether such reactions may be more frequent with modern SSRIs than with the older antidepressants such as tricyclics (TCAs) is still unclear. 

It is well-known that antidepressant treatment is associated with over-stimulation, restlessness resembling akathisia, agitation, insomnia, severe anxiety, mixed-dysphoric bipolar states, or psychosis in some patients [122,123,124,125,126]. Such paradoxical effects of antidepressant treatment have received attention in recent international discussions of risk/benefit considerations arising from studies of SSRIs, particularly when used for depressive disorders in adolescents. These discussions weigh the seemingly limited benefits of all types of antidepressant treatment for adolescent depression against concern about potential increases in suicidality [127]. 

The scientific and clinical significance of the effects of antidepressant treatment on suicidal risk requires further critical study, but our clinical impression is that antidepressant treatment may present certain behavioral risks that require close clinical monitoring, ongoing differential clinical assessment and adequate interventions in order to optimize the effectiveness and safety of antidepressant treatment. Several warning signs, including prominent anxiety and irritability, anger or agitation, or the appearance of these emotions early in the use of an antidepressant, particularly in antidepressant monotherapy, should be assessed and guide clinicians when treating patients with a high suicidal risk. Those with likely or suspected bipolar features or psychotic illnesses call for particular concern for potential behavioral risks under antidepressant monotherapy, without the presumably protective effects of mood-stabilizing or antipsychotic agents. 

## 8. The Adverse Effect of Antidepressants and Suicide Mortality

In 2004, two systematic reviews about the efficacy and safety data of antidepressants for children and adolescents were published simultaneously, one in *The British Medical Journal* [128] and the other in *The Lancet* [127]. Jureidini *et al*. [128] checked the quality of methods of reported published trials of antidepressants drugs in children and adolescents, involving a total of 477 patients from six randomized trials who were treated with paroxetine, fluoxetine, sertraline or venlafaxine and 464 treated with placebo. Of 42 reported outcome measures, only 14 showed a statistical advantage for antidepressant drugs. Regarding the adverse effects of treatment, the authors suggested that some adverse effects might be more frequent than the authors of the individual studies imply. For example, up to half of young patients experienced an “activation syndrome”, and self-injurious ideation was seen in 6% of patients [129]. However, this analysis did not investigate the relative frequency of this rare outcome. In the second review, Whittington *et al*. [127] reported no increased risk of suicidal behavior with fluoxetine compared to placebo (3.6% versus 3.8%), but an increased risk of suicidal ideation or attempting suicide was observed for paroxetine (3.7% versus 2.5%), sertraline (2.6% versus 1.2%), citalopram (7.1% versus 3.6%,) and venlafaxine (7.7% versus 0.6%). 

In a epidemiological study, Jick and collegues [71] followed 172,598 people who had at least one prescription for one of 10 antidepressant drugs in general practice in the UK. They found that the risk of suicide was higher in people who received fluoxetine (19 per 10,000 person years, 95%CI 9 to 34) than in those receiving dosulepine (dothiepin). In a nested case-controlled subgroup analysis in people with no history of suicidal behavior or previous antidepressant prescription, the risk remained the same, although the confidence interval broadened to make the result nonsignificant. However, the association between the adverse events in patients taking antidepressants and the risk of suicide was not reported. 

Jick and colleagues [72] performed a matched case-control study of more than 2,500 patients and reported that the risk of suicidal behavior after starting antidepressant treatment was similar among users of amitriptyline, fluoxetine, and paroxetine compared with the risk among users of dothiepin. The risk of suicidal behavior was similar in patients taking SSRIs and tricyclics, but dothiepin might not be a useful comparison antidepressant for countries where this agent has never been licensed, such as Italy and the USA [130].

To replicate this finding, Jick *et al*. [72] used amitriptyline as a reference compound, and the OR for non-fatal suicidal behaviors among users of the other three antidepressants were 1.21 (0.80–1.83) for dothiepin, 1.40 (0.92–2.13) for fluoxetine and 1.55 (0.99–2.43) for paroxetine. A borderline value for paroxetine in comparison with amitriptyline emerged in this post-hoc analysis, leaving the possibility of an increased risk of non-fatal suicidal behaviors in users of this agent.

To test the potential empirical link between antidepressant treatment and suicide attempts Valuck and colleagues [131] investigated 24,000 adolescents using a community sample of managed-care enrollees in the USA. Crude suicide attempt rates ranged from 0.0% to 2.3% by index treatment group. Treatment with SSRIs (hazard ratio 1.59), other antidepressant drugs (hazard ratio 1.03) or multiple antidepressants (hazard ratio 1.43) resulted in no significant increased risk of a suicide attempt. In contrast, Martinez *et al*. [80] analyzed the risk of non-fatal self-harm and suicide in 146,095 patients with a new diagnosis of depression who were prescribed SSRIs or tricyclics and reported that tricyclic users were not at increased risk of suicide or non-fatal self-harm. However, in patients aged 18 or less, weak evidence suggested a higher risk of non-fatal self-harm in those prescribed SSRIs (OR 1.59). Søndergård *et al*. [87] investigated the association between SSRIs and suicide in children and adolescents in Denmark and observed a statistically nonsignificant increased rate of suicide associated with SSRI use (RR 4.47). Simon *et al*. [84] identified more than 65,000 individuals with more than 82,000 episodes of antidepressant treatment. The risk of completed suicide during the acute-phase treatment was one in 3,000 treatment episodes, and the risk of a serious suicide attempt was one in 1,000. The data did not indicate a significant increase in risk of completed suicide or a serious suicide attempt after starting treatment with the newer antidepressants.

Isacsson *et al*. [59] analyzed the presence of different antidepressants in the forensic toxicological screening of 14,857 suicides compared with those in 26,422 cases of deaths by accident or natural causes in Sweden from 1992 to 2000. In the toxicological screening, antidepressants were detected in 3,096 (20%) of the 14,857 cases of suicide investigated and a total of 3,411 different antidepressants in these individuals. Of these, 48% were SSRIs. The number of detections in the 26,422 controls was 1,538. SSRIs were under-represented compared with other antidepressants (OR 0.83). The differences within the SSRIs were insignificant, with the exception of fluvoxamine. The OR for the tricyclics as a class (including maprotiline) was 1.01, but it was higher for the class of moclobemide, mianserin, mirtazapine, reboxetine and venlafaxine (OR 1.78, 99%CI 1.46–2.16). In 52 cases of suicide in those aged less than 15 years, seven were positive for antidepressants (clomipramine, imipramine, maprotiline, trimipramine, mianserin and venlafaxine), but no SSRIs were detected. In 326 cases of suicide in the 15 to 19 year-old group, there were 13 cases which were positive for antidepressants (amitriptyline, clomipramine, trimipramine, citalopram, fluoxetine, sertraline and mirtazapine), and SSRIs had lower RR in the suicides compared with non-SSRIs. Thus, the hypothesis that treatment of depressed individuals with SSRIs leads to an increased risk of suicide was not supported by this analysis of all suicides in Sweden over a period of 9 years.

In a nationwide cohort study in Finland, Tiihonen *et al*. [85] calculated the RR of completed suicide, suicide attempts leading to hospitalization and overall mortality during tricyclic, SSRI and SNRI treatment versus no antidepressant use, after adjusting for possible confounders. In the entire cohort, fluoxetine use (RR 0.52) was associated with a lower risk of suicide and venlafaxine (RR 1.61) with a higher risk. Among suicidal patients who had ever used antidepressants, the current use of any antidepressant was associated with a markedly increased risk of attempted suicide and with a markedly decreased risk of completed suicide and death. 

The relationship between venlafaxine use and suicidality was also investigated by Rubino *et al*. [132] who found that patients prescribed venlafaxine were more likely to complete or attempt suicide compared with patients prescribed citalopram, fluoxetine and dothiepin, but adjustment for a number of possible confounders substantially reduced the excess risk. Finally, Juurlink and colleagues [82] explored the relationship between the initiation of SSRIs and completed suicide in older patients. During the first month of therapy, SSRI utilization was associated with a nearly fivefold higher risk of completed suicide over the other antidepressants (adjusted odds ratio 4.8). The risk was independent of a recent diagnosis of depression or the receipt of psychiatric care, and suicides of a violent nature were more common during SSRI therapy. However, the absolute risk of suicide was low, maybe suggesting that the response to these agents may induce suicide only in a vulnerable subgroup of patients.

## 9. Conclusions

Research on the possible relationship between pharmacotherapy and suicidal behavior was virtually unknown until a decade ago. An initial case series and several reports, especially in the last ten years, indicated that a minority of depressed patients experience adverse behavioral and emotional responses to treatment with various antidepressant drugs, including SSRIs [38,123,133]. Some ecological studies reported minor decreases of suicide rates by region or over time associated with higher rates of prescribing modern antidepressants, but these correlations are presumably influenced by many factors, including access to clinical care. Most large clinical studies indicate decreased suicidal risk with antidepressant treatment, but others find increases for patients in some age groups. However, these studies often have confounding variables such as an association between antidepressant treatment and more severe depression and, thus, greater suicidal risk. Data pooled from relatively brief, randomized, controlled trials in patients with acute major depression suggest an increase in the risk of suicide attempts, as well as increases in suicidal ideation in younger patients and decreases in elderly patients. 

These data, taken together, suggest that antidepressants do not always have a beneficial effect on the risk of suicidal behavior, although they may reduce both suicidal ideation and other depressive symptoms. Peculiar responses, often emerging early in the course of antidepressant treatment, require close clinical follow-up in order to assess the risk of suicide. When this risk is present, treatment should be appropriately modified, for example, by changing antidepressant medication, adding sedating, antipsychotic or mood-stabilizing treatments, and providing additional individual support.

Suggested pharmacological interventions and other clinical steps aimed at safe management of patients at risk for the types of adverse behavioral responses to antidepressants have been described as follows. First, it is crucial to decrease the dose, or suspend antidepressant therapy, if agitation, insomnia or anger emerge. In addition, clinicians should introduce an atypical antipsychotic, anticonvulsant or sedative, and consider lithium for cooperative patients, especially following an inadequate antidepressant response. The collaborative and flexible nature of treatment required must emphasize the clinician’s availability for extra visits or contacts, especially in emergencies. Clinicians should express explicit concern for growing discomfort and despair; address suicidality directly and repeatedly; monitor for access to lethal means of self-injury; enlist the help of a family member to monitor the patient; and dispense appropriate medicines.

We conclude that antidepressant drugs generally appear to reduce suicidal ideation in depressed adults, but whether these agents impact suicidality in younger patients with major affective disorders is still a matter of debate. The possible increased suicide rate induced by the growing utilization of antidepressants remains one of the most important public health issues. Clinicians should be vigilant about the possible risk of iatrogenesis in prescribing potent drugs such as antidepressants [134]. The possible existence, particularly in younger patients, of many unrecognized pseudo-unipolar mixed states, which can be a clinical substrate for suicidality, may be one link that explains the increased suicide rate associated with the use of antidepressants in adolescent patients. 
